# Is age a predictor of mortality in medical high-dependency units?

**DOI:** 10.1186/cc9921

**Published:** 2011-03-11

**Authors:** E Hood, A Bhangu, D Pandit, A Michael

**Affiliations:** 1 Russells Hall Hospital, Dudley, UK

## Introduction

The population aged >65 years is set to rise by 32% by 2033. As resources are limited, difficult decisions regarding access to high-dependency care for the older person will become increasingly important. The aim of this study was to determine whether age is a predictor of mortality in patients admitted to an open medical high-dependency unit (MHDU).

## Methods

A prospective observational cohort study of 100 consecutive patients admitted to a MHDU with a medical diagnosis over a 3-month period. The primary endpoint was 30-day mortality.

## Results

**Figure 1:**
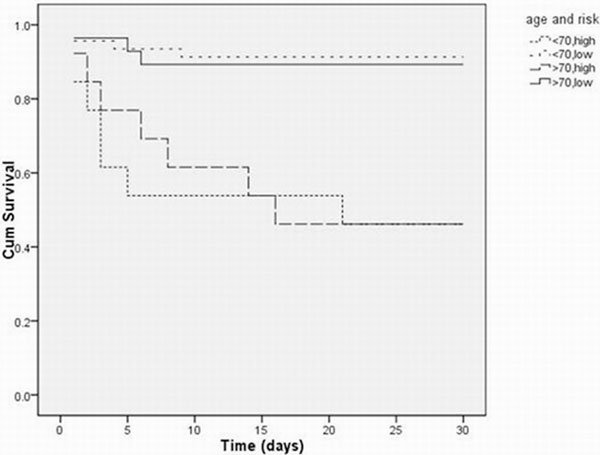
**Survival of high-risk (2+ organ support and high preadmission care levels) versus low-risk groups, split by age**.

Overall mortality at 30 days was 21% (*n *= 21). Forty-one per cent of patients were aged <65 years, 29% 65 to 74 years and 30% 75+ years. There were no significant differences in mortality between groups (12%, 31% and 23%, respectively). When considering APACHE II scores ≥25, there was no significant difference in mortality between age groups (35% <70 years (7/20) vs. 29% ≥70 years (4/14), *P *= 1.000). The final model at multivariable regression analysis identified that ≥2 organ support (odds ratio = 10.843, 95% CI = 3.281 to 35.836) and preadmission moderate/nursing home care (4.437, 95% CI = 1.053 to 18.697) were significantly associated with worse outcome. ROC curve analysis for death showed that APACHE II score was a moderate discriminator (area under the curve = 0.64, 95% CI = 0.53 to 0.75), and age (0.60, (0.48 to 0.72)) was a poor predictor for 30-day mortality. The majority of survivors (88%) were discharged at their preadmission functional status; those who declined in function were not significantly older than those who did not. See Figure 1.

## Conclusions

Age does not predict outcome from MHDU. Patients requiring ≥2 organ support and/or higher levels of preadmission home support had higher mortality. Selected elderly medical patients can be expected to have outcomes comparable with younger patients and should not be denied MHDU care.

